# The origin and development of subcortical U-fibers in gyrencephalic ferrets

**DOI:** 10.1186/s13041-020-00575-8

**Published:** 2020-03-10

**Authors:** Mayuko Yoshino, Kengo Saito, Kanji Kawasaki, Toshihide Horiike, Yohei Shinmyo, Hiroshi Kawasaki

**Affiliations:** Department of Medical Neuroscience, Graduate School of Medical Sciences, Kanazawa University, Takara-machi 13-1, Kanazawa, Ishikawa 920-8640 Japan

**Keywords:** Cerebral cortex, Ferret, In utero electroporation, Short association fibers, Subcortical U-fibers

## Abstract

In the white matter of the human cerebrum, the majority of cortico-cortical fibers are of short range, connecting neighboring cortical areas. U-fibers represent connections between neighboring areas and are located in the white matter immediately deep to the cerebral cortex. Using gyrencephalic carnivore ferrets, here we investigated the neurochemical, anatomical and developmental features of U-fibers. We demonstrate that U-fibers were derived from neighboring cortical areas in ferrets. U-fiber regions in ferrets were intensely stained with Gallyas myelin staining and Turnbull blue iron staining. We further found that U-fibers were derived from neurons in both upper and lower layers in neighboring areas of the cerebral cortex and that U-fibers were formed later than axons in the deep white matter during development. Our findings shed light on the fundamental features of U-fibers in the gyrencephalic cerebral cortex. Because genetic manipulation techniques for ferrets are now available, ferrets should be an important option for investigating the development, functions and pathophysiological changes of U-fibers.

## Introduction

In the white matter of the human cerebrum, the majority of cortico-cortical fibers are of short range, connecting neighboring cortical areas [1]. U-fibers, which serve as short-range association fibers, represent connections between neighboring areas of the cerebral cortex and are located in the white matter immediately deep to the cerebral cortex [1–5]. U-fibers are thought to mediate important higher-order cognitive functions of the nervous system [3, 5]. Consistently, while U-fibers are observed in both humans and monkeys [3, 6], they take up a larger proportion of the cerebrum in humans than they do in monkeys [7, 8].

Several human studies have suggested the involvement of U-fibers in neurological and psychiatric diseases. Subcortical U-fiber regions are among the predilection sites for multiple sclerosis [9–12]. Moreover, magnetic resonance imaging (MRI) and diffusion tensor imaging (DTI) studies have uncovered abnormalities in U-fiber regions in autism spectrum disorder [13, 14], schizophrenia [15, 16], Alzheimer’s disease [17] and progressive multifocal leukoencephalopathy patients [18–20]. Anatomical and neuroimaging techniques, including MRI and DTI, have been used for visualizing U-fiber regions in the brains of humans and monkeys [4, 21, 22]. However, because genetic manipulation techniques for monkeys are not well established, molecular mechanisms underlying the development, functions and diseases related to U-fibers are still largely unclear.

Recent pioneering studies using diffusion MR tractography reported that ferrets (*Mustela putorius furo*), like humans and monkeys, also have abundant U-fibers [23, 24]. Ferrets have a long history in research and have relatively well-developed brain structures such as cortical folds (i.e. gyri and sulci), which mice do not have. Ferrets have several advantages as model animals for neuroscientific research compared with monkeys. Usually, more than 6 ferret kits are born from one pregnant mother, and this large number of kits per pregnant mother enables us to make use of various experimental conditions and to obtain a sufficient number of experimental samples. Furthermore, we recently established rapid and simple genetic manipulation techniques for the cerebral cortex of ferrets [25–27]. Using these techniques, here we investigated the neurochemical, anatomical and developmental properties of U-fibers using ferrets. Ferrets are an intriguing new option for investigating the development, functions and pathophysiological changes of U-fibers.

## Results

**Fig. 1:**
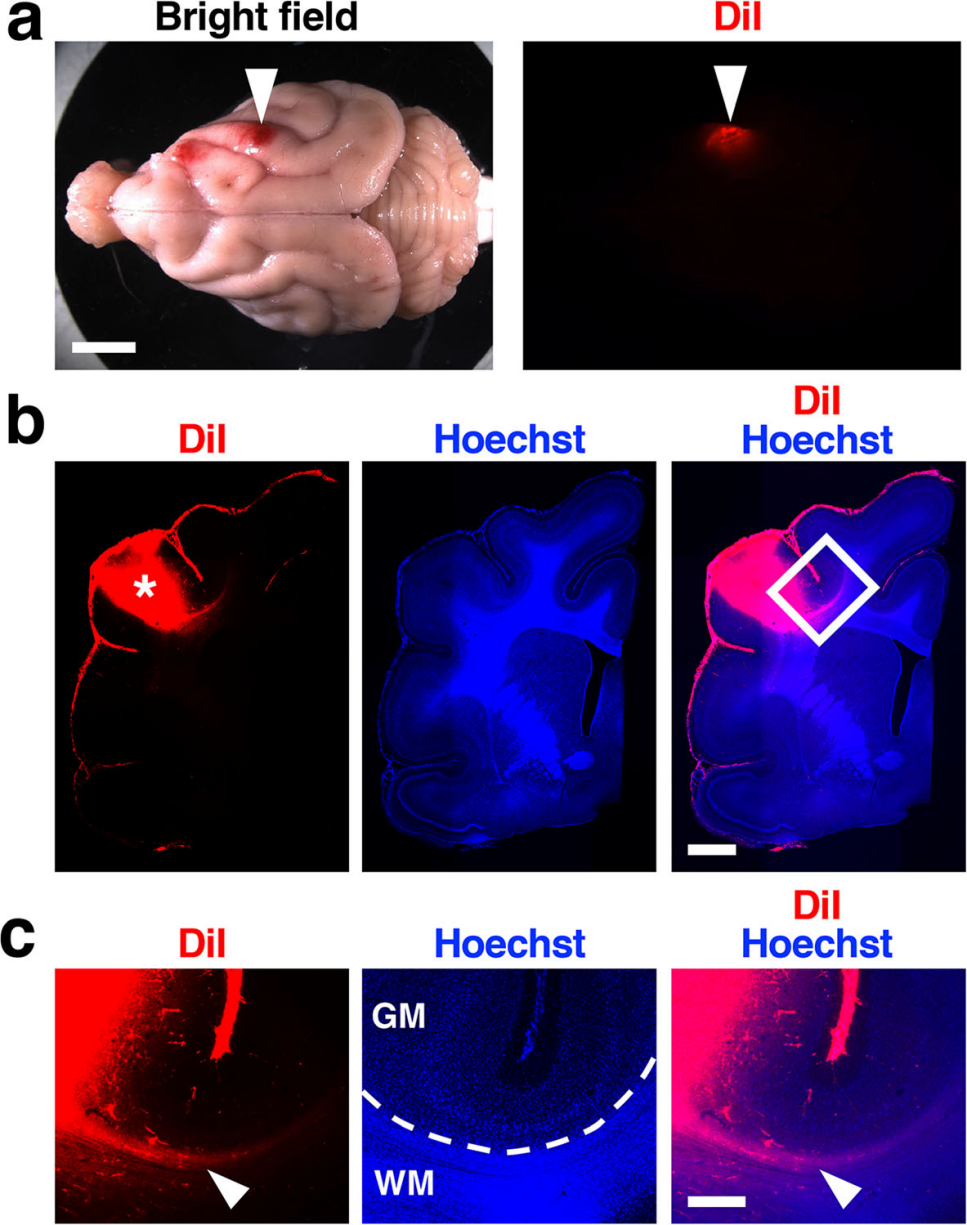
U-fibers labeled with DiI in the adult ferret brain. **a** A DiI crystal was inserted into the cerebral cortex of the adult ferret. Dorsal views of the brain are shown. The arrowheads indicate the position of the DiI crystal. **b** Coronal sections of the DiI-implanted brain. The asterisk indicates the position of a DiI crystal. **c** Magnified images of the boxed area in (**b**). DiI-positive fibers were observed in the white matter immediately deep to the cerebral cortex (arrowheads). The broken line indicates the border between the gray matter and the white matter. GM, gray matter; WM, white matter. Scale bars = 1 cm (**a**), 2 mm (**b**), 500 μm (**c**)

U-fibers are short-range association fibers connecting neighboring cortical areas in humans [1, 3]. We therefore examined whether U-fibers are also derived from neighboring cortical areas in ferrets. We injected the lipophilic tracer DiI into the cerebral cortex of living adult ferrets (Fig. 1a, arrowheads). We then prepared brain samples 2 days later and made coronal sections of the cerebrum (Fig. 1b). We found DiI-positive fibers running immediately deep to the cerebral cortex (Fig. 1c, arrowheads), suggesting that ferret U-fibers are derived from cortical neurons nearby, as is the case with human U-fibers.

**Fig. 2:**
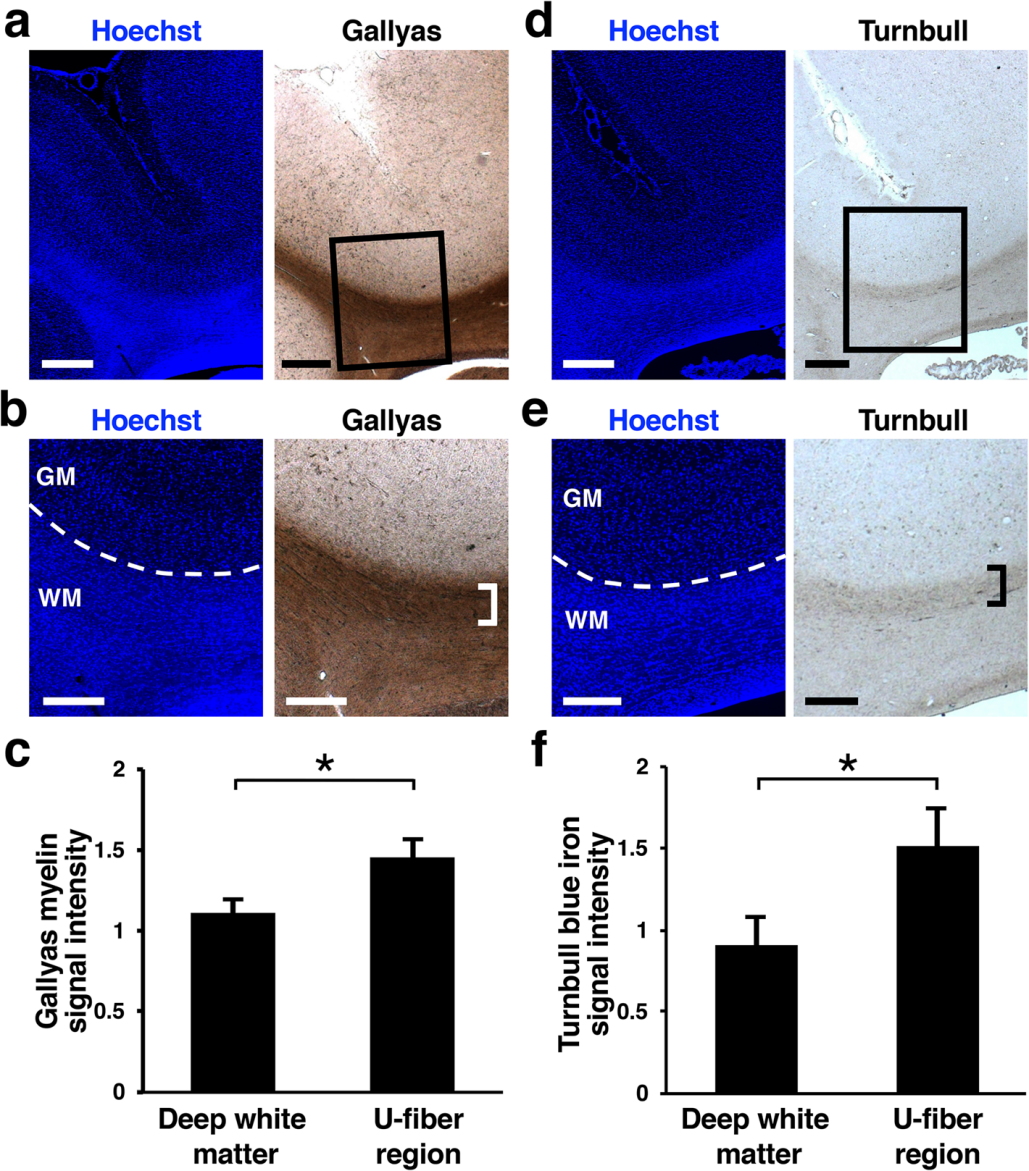
Gallyas myelin staining and Turnbull blue iron staining using sections of adult ferrets. **a**, **b** Gallyas myelin staining. **a** Lower magnification images of the cerebral cortex. **b** Higher magnification images of the boxed area in (**a**). Note that the U-fiber region was densely labeled (square bracket). **c** Quantification of staining intensities of Gallyas myelin staining. Staining intensities of U-fiber regions were significantly higher than those in the deep white matter. Bars present mean ± SD. *p < 0.05; Welch’s *t*-test; *n* = 3 animals. **d**, **e** Turnbull blue iron staining. **d** Lower magnification images of the cerebral cortex. **e** Higher magnification images of the boxed area in (**d**). Note that the U-fiber region was densely labeled (square bracket). **f** Quantification of staining intensities of Turnbull blue iron staining. Staining intensities of U-fiber regions were significantly higher than those in the deep white matter. Bars present mean ± SD. *p < 0.05; Welch’s *t*-test; *n* = 3 animals. GM, gray matter; WM, white matter. Scale bars = 500 μm (**a**, **d**) and 300 μm (**b**, **e**)

We next examined whether ferret U-fibers and human U-fibers share similar neurochemical properties. Because it was reported that human and monkey U-fiber regions were densely labeled with Gallyas myelin staining [28, 29], we performed Gallyas myelin staining using coronal sections of the adult ferret brain. As is also the case in the human brain, U-fiber regions were strongly labeled with Gallyas myelin staining in the ferret brain (Fig. 2a and b, square bracket), suggesting that myelin is densely accumulated in U-fiber regions of the ferret brain, and that ferret U-fibers and human U-fibers share similar characteristics. Consistent with this observation, our quantification showed that of Gallyas myelin staining intensities in U-fiber regions were significantly higher than those in the deep white matter (deep white matter, 1.10 ± 0.09; U-fiber region, 1.45 ± 0.12; Welch’s *t*-test, *p < 0.05) (Fig. 2c). Another study reported that iron was accumulated at the cortico-subcortical junction in the human brain [30]. We therefore performed Turnbull blue iron staining using coronal sections of the adult ferret brain and found an accumulation of iron in U-fiber regions (Fig. 2d and e, square bracket). Consistently, our quantification showed that Turnbull blue iron staining intensities in U-fiber regions were significantly higher than those in the deep white matter (deep white matter, 0.91 ± 0.17; U-fiber region, 1.52 ± 0.23; Welch’s *t*-test, *p < 0.05) (Fig. 2f). Because iron is mainly stored in ferritin in myelin [30], it seems likely that the accumulation of iron in the U-fiber regions results from myelin accumulation in the U-fiber regions. These results suggest that iron accumulation is another feature of the U-fiber regions.

**Fig. 3:**
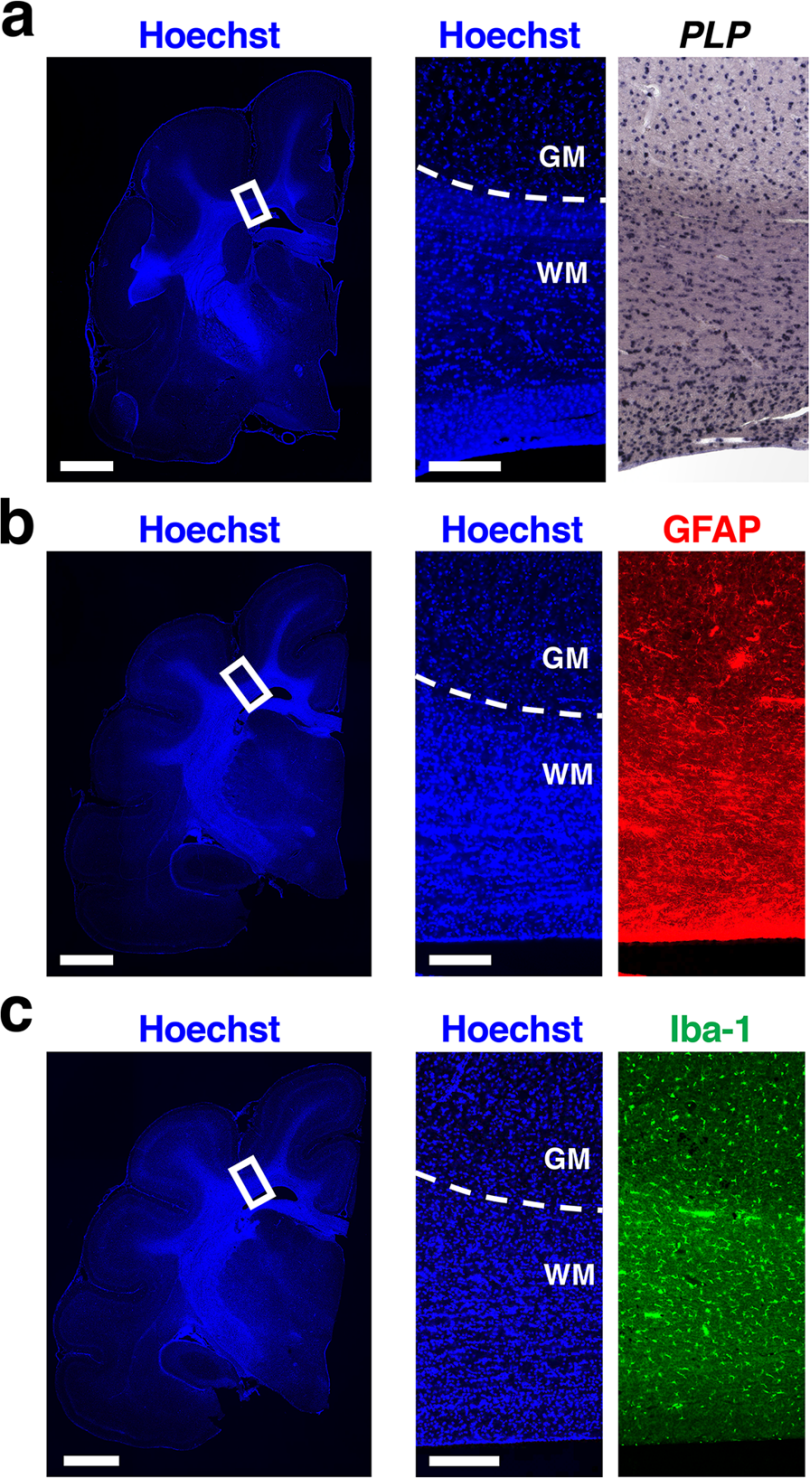
Distribution patterns of oligodendrocytes, astrocytes and microglia around the white matter. In situ hybridization for *PLP* (**a**) and immunohistochemistry for GFAP (**b**) and Iba-1 (**c**) were performed using brain sections of adult ferrets. Lower magnification Hoechst images are shown on the left. In situ hybridization, immunostaining and Hoechst images corresponding to the boxed areas in the lower magnification images are shown on the right. Note that oligodendrocytes, astrocytes and microglia were similarly distributed in the U-fiber region and the deep white matter. GM, gray matter; WM, white matter. Scale bars = 2 mm (left) and 200 μm (right)

Because myelin was accumulated in U-fiber regions, we next performed in situ hybridization for *PLP*, which labels oligodendrocytes, using coronal sections of the adult ferret brain. Unexpectedly, we found that oligodendrocytes were similarly distributed in the U-fiber region and the deep white matter in each section (Fig. 3a). This result excludes the possibility that dense labeling of U-fiber regions by Gallyas myelin staining resulted from an abundance of oligodendrocytes in U-fiber regions. It therefore seems plausible that myelin sheaths in U-fiber regions are more developed than those in the deep white matter. In addition, we also performed immunohistochemistry for GFAP and Iba-1, which are expressed in astrocytes and microglia, respectively. GFAP-positive cells and Iba-1-positive cells were similarly distributed in the U-fiber region and the deep white matter in each section (Fig. 3b and c), suggesting that astrocytes and microglia do not exhibit specific distribution patterns in the white matter of the ferret cerebrum.

**Fig. 4:**
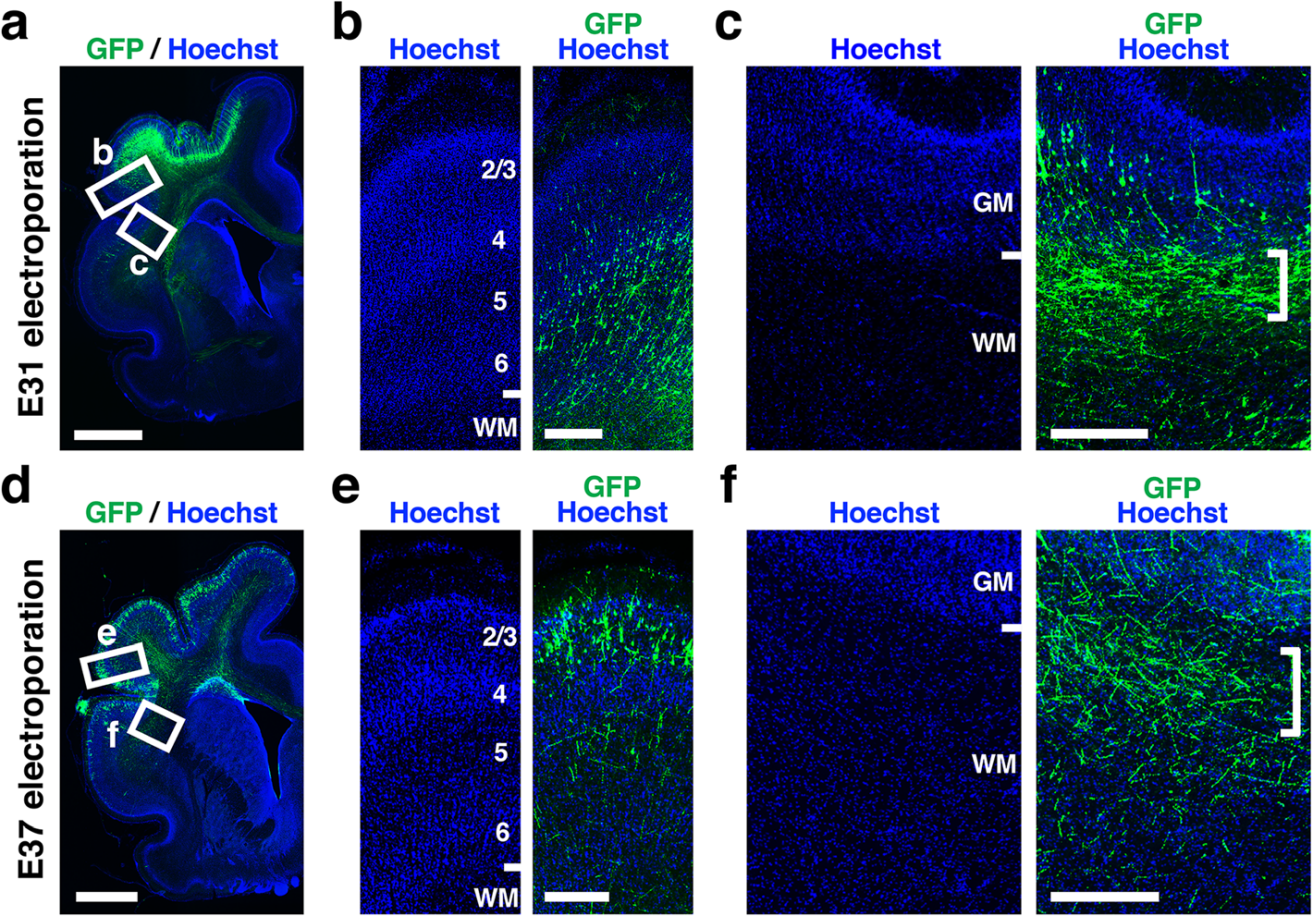
Cellular origin of U-fibers revealed with in utero electroporation. GFP was introduced into lower-layer neurons and upper-layer neurons using in utero electroporation at E31 (**a**-**c**) and E37 (**d**-**f**), respectively. Coronal sections were prepared at P16. **a**, **d** Lower magnification images of the coronal sections. The boxed areas were magnified and are shown in (**b**, **c**, **e**, **f**). **b**, **e** Higher magnification images of the cerebral cortex. **c**, **f** Higher magnification images of the white matter. At both E31 and E37, GFP-positive fibers were observed in the white matter immediately deep to the cerebral cortex (square brackets). Numbers indicate layers in the cerebral cortex. GM, gray matter; WM, white matter. Scale bars = 2 mm (**a**, **d**) and 300 μm (**b**, **c**, **e** and **f**)

We next investigated from which layers of the cerebral cortex U-fibers are derived. For this purpose, we electroporated the GFP-expressing plasmid into the ferret cerebral cortex at different developmental stages (Fig. 4) [25–27]. In utero electroporation was performed at either E31 or E37, and coronal sections were prepared from the middle third of the cerebrum at P16. Consistent with our previous report [25], in utero electroporation at E31 and E37 introduced GFP into lower-layer and upper-layer neurons, respectively (Fig. 4a, b, d and e). We found GFP-positive U-fibers in both cases (Fig. 4c and f, square brackets), suggesting that both lower- and upper-layer neurons contribute to U-fibers in ferrets.

**Fig. 5:**
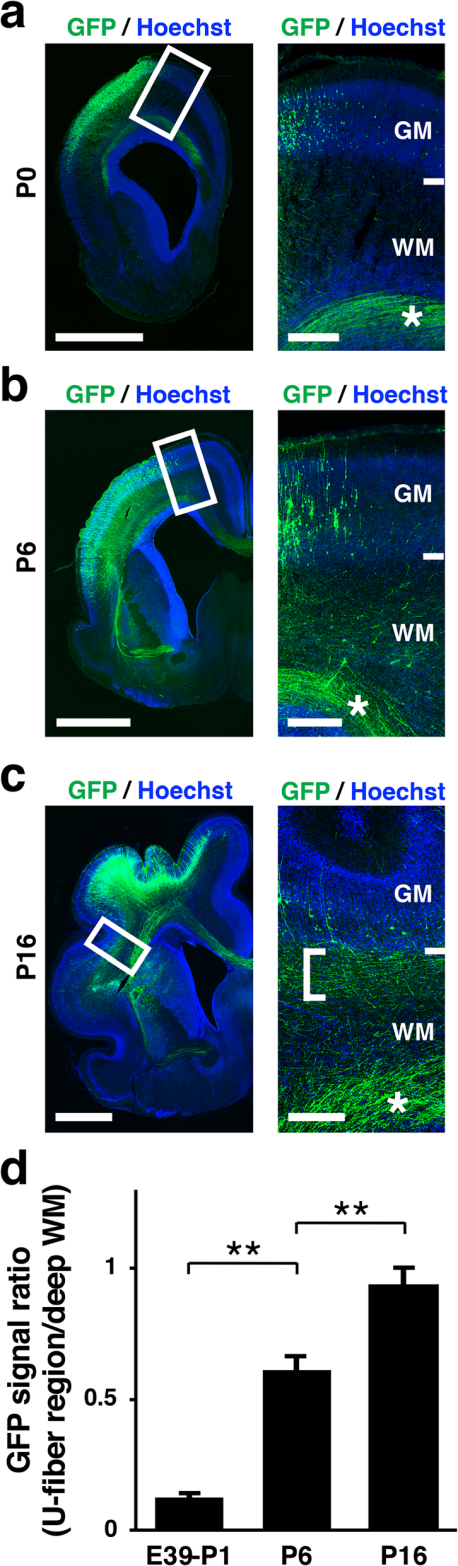
Development of U-fibers revealed with in utero electroporation. GFP was introduced into cortical neurons using in utero electroporation at E31, and coronal sections were prepared at P0 (**a**), P6 (**b**) and P16 (**c**). Lower magnification images are shown on the left. Higher magnification images of the boxed areas are shown on the right. Note that GFP-positive fibers in the white matter immediately deep to the cerebral cortex were rarely observed at P0 and increased between P0 and P16 (square bracket). In contrast, abundant GFP-positive fibers in the deep white matter were clearly visible even at P0 (asterisks). **d** The ratios of GFP signal intensities in the U-fiber regions relative to those in the deep white matter. The ratios of GFP signals in the U-fiber regions to those in the deep white matter significantly increased between P1 and P16. Bars present mean ± SD. **p < 0.01; Welch’s *t*-test; n = 3 animals for each condition. GM, gray matter; WM, white matter. Scale bars = 2 mm (left) and 300 μm (right)

Although the developmental processes of U-fibers have been examined using DTI and histological techniques [5], developmental processes at the single axon level remained to be investigated. We therefore visualized U-fibers by expressing GFP using our in utero electroporation techniques for ferrets. In utero electroporation was performed at E31 to introduce GFP into cortical neurons, and coronal sections were prepared from the middle third of the cerebrum at P0, P6 and P16. Interestingly, we found that GFP-positive fibers in the white matter just under the cerebral cortex were rarely found at P0 (Fig. 5a) and increased between P0 and P16 (Fig. 5c, right panel, square bracket). In contrast, abundant GFP-positive fibers in the deep white matter were clearly observed even at P0 (Fig. 5a-c, right panels, asterisks). Consistent with these observations, the ratios of GFP signals in the U-fiber region relative to those in the deep white matter significantly increased between P1 and P16 (E39-P1, 0.12 ± 0.01; P6, 0.61 ± 0.05; P16, 0.94 ± 0.06; Welch’s *t*-test, **p < 0.01) (Fig. 5d). These results indicate that U-fibers are formed neonatally after axons in the deep white matter are formed. Because U-fibers and deep white matter axons are formed at different time points, it seems plausible that the development of U-fibers is regulated in a different manner from that of deep white matter axons. The mechanisms of axon guidance and elongation could differ between U-fibers and deep white matter axons.

## Discussion

Here we have demonstrated that U-fiber regions in ferrets were intensely labeled with Gallyas myelin staining and Turnbull blue iron staining. U-fibers were derived from neurons in both upper and lower layers in neighboring areas of the cerebral cortex and were formed later than axons in the deep white matter during development. Our results indicate that ferrets are an intriguing new option for investigating the development, functions and pathophysiological changes of U-fibers.

Our findings using in utero electroporation demonstrated that U-fibers are derived from both supragranular and infragranular layers of the cerebral cortex in ferrets. According to previous literature, short association fibers originating from the supragranular layers are considered to transfer information in a forward direction, whereas feedback connections originate from the infragranular layers [3]. It seemed likely that both feedforward and feedback connections of U-fibers were visualized with GFP transfection in the ferret cerebral cortex. It would be intriguing to investigate which layers of the cerebral cortex the axons of U-fibers project to. U-fibers derived from the supragranular layers and those derived from the infragranular layers may project to different layers in the neighboring cerebral cortex. Co-transfection of GFP plus synaptophysin-mCherry, which labels presynaptic terminals [31], would be useful for addressing this point. In addition, we demonstrated that U-fibers are formed later than axons in the deep white matter during development. This seems consistent with previous studies showing that U-fibers become myelinated late during development [32, 33].

Previously, anatomical and neuroimaging techniques have been used for investigating U-fiber regions in humans, monkeys, ferrets and mice. These pioneering studies demonstrated that humans have larger U-fiber regions than monkeys [7, 8] but failed to identify U-fibers in mice [23]. These studies also showed that U-fibers are involved in various neurological and psychiatric diseases in humans [9–20]. Interestingly, it was reported that U-fiber regions have dual blood supply, which may lead to unique protection from ischemic events [34]. Although these studies have provided important information about U-fibers, genetic investigations of U-fibers were lacking. In this study, using our in utero electroporation technique for ferrets, we found that U-fibers were derived from neurons in both upper and lower layers in neighboring areas of the cerebral cortex and were formed later than axons in the deep white matter during development. For further investigations into the mechanisms underlying the development, functions and diseases of U-fibers, combining research on humans, monkeys and ferrets would be important, as each has its own advantages. In addition, although U-fibers have not been identified in mice, it would be intriguing to investigate the evolutionary origin of U-fibers using mice.

Although it has been proposed that U-fibers mediate important cognitive functions [3, 5], their precise roles in information processing and animal/human behaviors are still unclear. Similarly, although several studies reported the developmental and maturation processes of U-fibers, the molecular mechanisms responsible for these processes are largely unknown. Furthermore, the pathophysiological involvement of U-fiber lesions in various kinds of diseases remains to be elucidated. Combining ferrets and our genetic manipulation techniques would contribute to our understanding of the entire picture of the development, functions and diseases of U-fibers.

## Methods

### Animals

Normally pigmented sable ferrets (*Mustela putorius furo*) were purchased from Marshall Farms (North Rose, NY). Ferrets were maintained as described previously [35–37]. The day of conception and that of birth were counted as embryonic day 0 (E0) and postnatal day 0 (P0), respectively. All procedures were performed in accordance with a protocol approved by the Animal Care Committee of Kanazawa University. All experiments were performed at least 3 times and gave consistent results.

### In utero electroporation procedure for ferrets

The in utero electroporation procedure for expressing transgenes in the ferret brain was described previously [25, 26]. Briefly, pregnant ferrets were anesthetized, and their body temperature was monitored and maintained using a heating pad. The uterine horns were exposed and kept wet by adding drops of phosphate-buffered saline (PBS) intermittently. The location of embryos was visualized with transmitted light delivered through an optical fiber cable. The pigmented iris was visible, and this enabled us to assume the location of the lateral ventricle. Approximately 2–5 μl of DNA solution was injected into the lateral ventricle at the indicated ages using a pulled glass micropipette. Each embryo within the uterus was placed between tweezer-type electrodes with a diameter of 5 mm (CUY650-P5; NEPA Gene, Japan). Square electric pulses (50–100 V, 50 ms) were passed 5 times at 1-s intervals using an electroporator (ECM830, BTX). The wall and skin of the abdominal cavity were sutured, and the embryos were allowed to develop normally.

### Plasmids

pCAG-EGFP was described previously [38]. Plasmids were purified using the Endofree Plasmid Maxi Kit (Qiagen, Valencia, CA). Prior to in utero electroporation procedures, plasmid DNA was diluted in PBS, and Fast Green solution was added to a final concentration of 0.5% to monitor the injection.

### Immunohistochemistry

Immunohistochemistry was performed as described previously with slight modifications [39, 40]. Briefly, ferrets were deeply anesthetized and transcardially perfused with 4% paraformaldehyde (PFA). The brains were dissected and post-fixed overnight with 4% PFA in PBS. The brains were cryoprotected by three-day immersion in 30% sucrose and embedded in OCT compound. Coronal sections of 50 μm thickness were prepared from the middle third of the cerebrum using a cryostat. Then the sections were permeabilized with 0.3% Triton X-100 in PBS and blocked with 2% skim milk, 0.3% Triton X-100 in PBS. The sections were then incubated overnight with primary antibodies, which included anti-GFAP (Sigma-Aldrich, G3893), anti-Iba1 (Wako, 019–19741) and anti-GFP antibodies (Medical & Biological Laboratories, 598). After incubation with secondary antibodies and Hoechst 33342, the sections were washed and mounted.

### In situ hybridization

In situ hybridization was performed as described previously with modifications [41, 42]. Sections of 30 μm thickness were prepared from fixed tissues using a cryostat and were treated with Hoechst 33342. Then the sections were treated with 4% PFA for 10 min, 1 μg/ml proteinase K for 10 min and 0.25% acetic anhydride for 10 min. After prehybridization, the sections were incubated overnight at 58 °C with digoxigenin-labeled RNA probes diluted in hybridization buffer (50% formamide, 5x SSC, 5x Denhardt’s solution, 0.3 mg/ml yeast RNA, 0.1 mg/ml herring sperm DNA, and 1 mM dithiothreitol). The sections were then incubated with alkaline phosphatase-conjugated anti-digoxigenin antibody (Roche, 11093274910) and Hoechst 33342, and were visualized using NBT/BCIP as a substrate. To make the *PLP* probe, the ferret *PLP* gene was amplified with RT-PCR and inserted into the EcoRI site of the pCRII vector. The primers used were as follows: forward, attgaattcagtcagagttccaaagacatg; reverse, aattgaattctcagaacttggtgcctcg.

### DiI tracing

After adult ferrets were deeply anesthetized, a skin incision was made, and temporal muscles on the skull were removed using an electrical scalpel. After small regions of the skull were removed, a small piece of DiI crystal (Molecular Probes, D282) was inserted into the cerebral cortex. Two days later, the ferrets were transcardially perfused with 4% PFA. Sections of 100 μm thickness were made using a vibratome and stained with Hoechst 33342.

### Gallyas myelin staining

Gallyas myelin staining was performed as described previously with slight modifications [43, 44]. To prepare an ammoniacal silver nitrate solution, 0.01 g of ammonium nitrate and 0.01 g of silver nitrate were dissolved in 10 mL water, and 0.03 ml of 4% sodium hydroxide was added, and the pH of the solution was adjusted to 7.5. To prepare the developing solution, solution A (0.5 g anhydrous sodium carbonate in 10 mL water) and solution B (0.02 g ammonium nitrate, 0.02 g silver nitrate and 0.1 g of tungstosilicic acid in 10 mL water) were made. The developing solution was made by mixing 10 mL of solution A, 10 mL of solution B and 73 μl of 4% PFA by stirring immediately before use.

Sections of 50 μm thickness were prepared from fixed tissues using a cryostat and were treated with 10% formalin for 1 month. The sections were stained with Hoechst 33342, and Hoechst images were taken. The sections were then immersed in a 2:1 mixture of pyridine and acetic anhydride for 30 min and washed in 80% pyridine, 60% pyridine, 40% pyridine, 20% pyridine and then water. The sections were incubated in the ammoniacal silver nitrate solution for 45 min. After being washed with 0.5% acetic acid three times, the sections were treated with 10% formalin for 2–5 days. After being washed with 0.5% acetic acid, the sections were immersed in the developing solution for 15–60 min and were washed with 1% acetic acid to terminate the color reaction. The sections were immersed in 0.1% potassium ferricyanide for a few tens of seconds to increase the contrast. Finally, the sections were washed three times with water.

### Turnbull blue iron staining

Turnbull blue iron staining was performed as described previously with slight modifications [45, 46]. Sections of 30 μm thickness were prepared from fixed tissues using a cryostat. The sections were treated with Hoechst 33342, and Hoechst images were taken. The sections were then incubated with 0.5% HCl and 5% potassium ferricyanide for 15 min and washed with PBS. The sections were then incubated in methanol containing 0.3% H_2_O_2_ and 0.01 M NaN_3_ for 30 min to inhibit peroxidase and catalase activities. After being washed with 0.1 M phosphate buffer at pH 7.4, the sections were treated with 0.1 M phosphate buffer containing 0.025% DAB, 0.005% H_2_O_2_ and 0.005% CoCl_2_. Finally, the sections were washed three times with PBS.

### Microscopy

Epifluorescence microscopy was performed with a BIOREVO BZ-9000 (Keyence) and an Axioimager A1 microscope (Carl Zeiss). Bright-field images were taken with an MZ16F fluorescence stereomicroscope (Leica), a BIOREVO BZ-9000 (Keyence) and an Axioimager A1 microscope (Carl Zeiss).

### Quantitative analyses

For quantification of Gallyas myelin staining and Turnbull blue iron staining, coronal sections from the middle third of the cerebrum were prepared from 3 animals. From each section, staining intensities of 3 points of the U-fiber region, 3 points in the deep white matter and 3 points in the corpus callosum were measured using ImageJ software, and average values were calculated. For each section, after background staining intensity was subtracted, the ratio of the average signal intensity in the U-fiber region to that in the corpus callosum and the ratio of the average signal intensity in the deep white matter to that in the corpus callosum were calculated for normalization.

For quantification of GFP signals, coronal sections from the middle third of the cerebrum were prepared from 3 animals. From each section, GFP signal intensities in 3 points of the U-fiber region and in 3 points of the deep white matter were measured using ImageJ software, and average values were calculated. For each section, after background signal intensity was subtracted, the average value of the U-fiber region was divided by that of the deep white matter.

## Data Availability

The datasets used and/or analyzed during the current study are available from the corresponding author on request.

## Abbreviations

DTI: Diffusion tensor imaging
GM: Gray matter
MRI: Magnetic resonance imaging
PBS: Phosphate-buffered saline
PFA: Paraformaldehyde
WM: White matter
